# Supplementary material for: Predicting visual function by interpreting a neuronal wiring diagram
Source: Nature. 2024 Oct 2;634(8032):113–23. doi: 10.1038/s41586-024-07953-5 (PMC11446822; doi:10.1038/s41586-024-07953-5)
Supplement: Supplementary file 6 — CRF and ERF predictions for individual TmY4 and TmY9 cells. Analogous to Supplementary Data 3, but for TmY target types. Shown are the top four monosynaptic pathways, the strongest pathway passing through each of the top ten intermediary types (ranking from Extended Data Fig. 7), and the trisynaptic pathway Tm1–TmY–Dm3–TmY (see the section entitled Prediction of spatial normalization). [file 41586_2024_7953_MOESM6_ESM.zip › DataS4/TmY9qΓèÑ/720575940620183888.pdf]

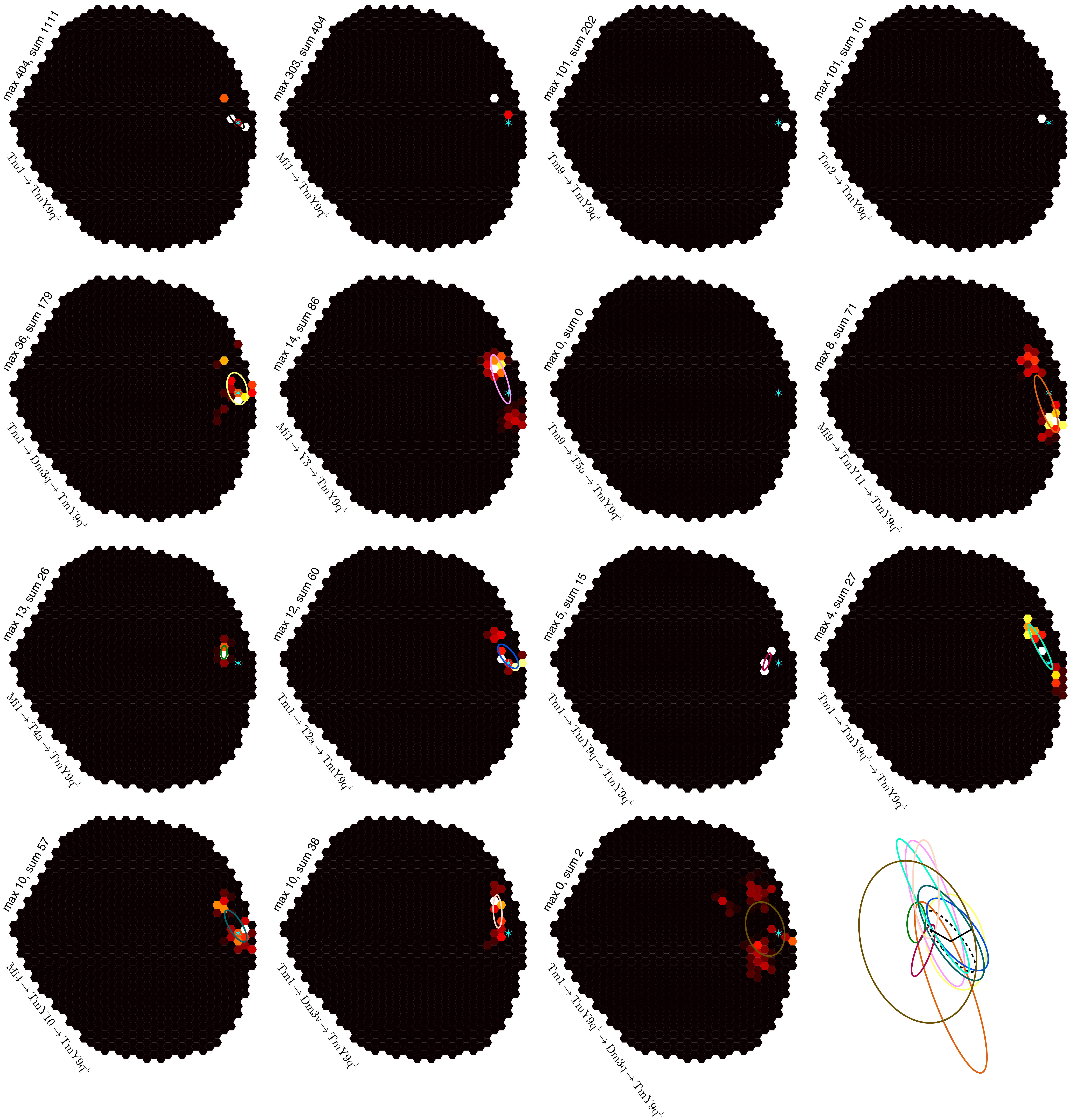

max 404, sum 1111
Tm1 → TmY9q⊥
max 303, sum 404
Mi1 → TmY9q⊥
max 101, sum 202
Tm9 → TmY9q⊥
max 101, sum 101
Tm2 → TmY9q⊥
max 36, sum 179
Tm1 → Dm3q → TmY9q⊥
max 14, sum 86
Mi1 → Y3 → TmY9q⊥
max 0, sum 0
Tm9 → T5a → TmY9q⊥
max 8, sum 71
Mi9 → TmY11 → TmY9q⊥
max 13, sum 26
Mi1 → T4a → TmY9q⊥
max 12, sum 60
Tm1 → T2a → TmY9q⊥
max 5, sum 15
Tm1 → TmY9q → TmY9q⊥
max 4, sum 27
Tm1 → TmY9q⊥ → TmY9q⊥
max 10, sum 57
Mi4 → TmY10 → TmY9q⊥
max 10, sum 38
Tm1 → Dm3v → TmY9q⊥
max 0, sum 2
Tm1 → TmY9q⊥ → Dm3q → TmY9q⊥
